# Correlation between hearing impairment and the Triglyceride Glucose Index: based on a national cross-sectional study

**DOI:** 10.3389/fendo.2023.1216718

**Published:** 2023-06-22

**Authors:** Long Liu, Maolin Qin, Jiabiao Ji, Weiqing Wang

**Affiliations:** ^1^ Department Otorhinolaryngology, Head and Neck Surgery, The Second Affiliated Hospital of Anhui Medical University, Hefei, Anhui, China; ^2^ Department Otorhinolaryngology, Head and Neck Surgery, Wuhu Hospital, East China Normal University (The Second People’s Hospital of Wuhu), Wuhu, Anhui, China

**Keywords:** insulin resistance, triglyceride glucose index, hearing impairment, cross-sectional study, NHANES

## Abstract

**Objective:**

Data from the National Health and Nutrition Examination Survey (NHANES) were used to assess the association between the triglyceride-glucose (TyG) index and hearing impairment (HI).

**Methods:**

We used eight survey cycles from NHANES 2001–2012 and 2015–2018 to conduct this cross-sectional study. HI was designed as an dependent variable, and the TyG index was selected as an exposure factor (independent variable). The correlation between the two variables was assessed using multiple logistic regression. In order to assess whether there was a non-linear relationship between the TyG index and HI, the TyG index was distributed and a test for trend was conducted (P for trend), followed by smooth curve fitting (penalized spline) and generalized additive model (GAM) regression. We also performed a subgroup analysis to identify sensitive groups whose responses were clearly associated with independent variables.

**Results:**

10,906 participants were finally included in the study, and those with a higher TyG index had a higher frequency of hearing impairment. There was a linear positive correlation between the TyG index and HI. For the low-frequency HI, however, this positive correlation was not statistically significant (OR = 1.05, 95% CI: 0.98, 1.14); however, it was more stable for the high-frequency HI (OR = 1.12, 95% CI: 1.03, 1.22). Additionally, as the TyG index increased, this positive association increased as well (P for trend = 0.05). The HPTA test showed a positive association with more severe HI (simultaneous) as the independent variable increased (OR = 1.14, 95% CI: 1.05–1.24), and this association was even more significant with increasing severity (P for trend 0.05). According to the subgroup analysis, the positive association between TyG index and high-frequency HI was more significant in females, 40–69 years old, without hypertension or diabetes, and when strict high-frequency HI was significant in males, females, 40–69 years old, with hypertension and diabetes.

**Conclusion:**

Participants with a higher TyG index may have a higher risk of HI. TyG index and HI risk showed a linear relationship, which became even more significant when HPTA was included.

## 1 Introduction

In the world, hearing impairment (HI) is ranked fourth among the leading causes of disability (1), and its global burden increases with age. According to previous studies, about half of people between the ages of 60 and 69 were believed to be affected by HI. As early as the age of 80, up to 80% of patients would suffer from HI, and the damage caused by it becomes more evident (2, 3). The incidence of HI increases significantly after the age of 15 as well as among the elderly (4). According to previous studies, hearing loss imposed an enormous burden on society and the economy. When the annual monetary loss from hearing loss is calculated, it exceeds US$750 billion (5). However, screening for HI is not common in everyday life. HI screening is often ignored because of its high cost and because patients perceive it as unnecessary. Therefore, many patients have difficulty accepting effective and timely treatment (6). A lot of attention has been paid to research into the factors that increase the risk of HI as well as the development of timely and effective prevention measures.

It is not possible to identify the cause of HI from a theoretical standpoint, as any part of the central auditory nervous system can cause hearing loss. Ageing, genetic mutations, noise exposure, therapeutic drug exposure with ototoxic side effects, and chronic disease degradation processes are the most important risk factors (6). It has been recognized by researchers that diabetes may lead to HI as one of several risk factors (7). Generally, diabetes is more expensive to our health care system than most other health outcomes, including the impact that diabetes has on accelerated natural aging. Numerous studies have shown that many diseases, such as diabetes complications, are actually affected by insulin resistance (IR) before diabetes has even begun (8–10). In spite of this, very little research has been conducted on the relationship between insulin resistance and HI as of this writing. There was a positive correlation between HI and the insulin homeostasis model (HOMA-IR) in SeoM (11). However, due to the small number of people with diabetes who use insulin, HI and IR need to be further explored.

A hyperinsulinemic normoglycaemic clamp (HEC) is the gold standard for measuring insulin, but this is difficult in a non-study setting (12). In previous studies, HOMA-IR has been widely used as a noninvasive method for studying IR, but it is insulin-dependent and of limited value for subjects on insulin therapy or with no functional -cells (13). Consequently, new and effective assessments of IR levels must be developed. During the past decade, researchers have been interested in the triglyceride-glucose (TyG) index. This index was shown to be superior to the HOMA-IR model for assessing insulin resistance in diabetic and non-diabetic individuals (14).For example, Guerrero-Romero F demonstrated that an optimal value of 4.68 for the TyG index showed the highest sensitivity (96.5%) and specificity (85.0%); the area under the curve was able to reach 0.858 (15). Vasques AC explored the correlations between TyG index and HOMA-IR with insulin resistance from the hyperglycemic clamp test in a study of a Brazilian population. The results showed that the TyG index had a higher correlation with insulin resistance compared to HOMA-IR, with Pearson correlation coefficients of -0.64 and -0.51, respectively.In addition, the TyG index had better diagnostic power for IR (AUC=0.79) (16). In addition, the advantages of the TyG index have been demonstrated in many diseases, such as cardiovascular disease (17), type 2 diabetes (18) and polycystic ovary syndrome (PCOS) (19). In spite of this, no studies have been conducted on how the TyG index correlates with HI. To summarize, the aim of the study was to determine the correlation between the TyG index and the HI based on a cross-sectional study of the National Health and Nutrition Examination Survey (NHANES) cohort.

## 2 Materials and methods

### 2.1 Study population

Throughout this study, data were drawn from the NHANES database. To obtain large data sets representative of the US population, the NHANES survey used a complex multistage probability sampling design. There were six sections to the survey, including sociodemographic information, dietary intake and habits, health behaviors, medical history, and physiological and laboratory examinations. The Research Ethics Review Board of the National Center for Health Statistics (NCHS) reviewed and standardized the NHANES protocols each year in compliance with the Human Research Subjects Protection Policy of the US Department of Health and Human Services (HHS). The consent of all participants was obtained in a verbal and written manner. The NHANES freed up all the data without further permission or ethical review.

**Figure 1:**
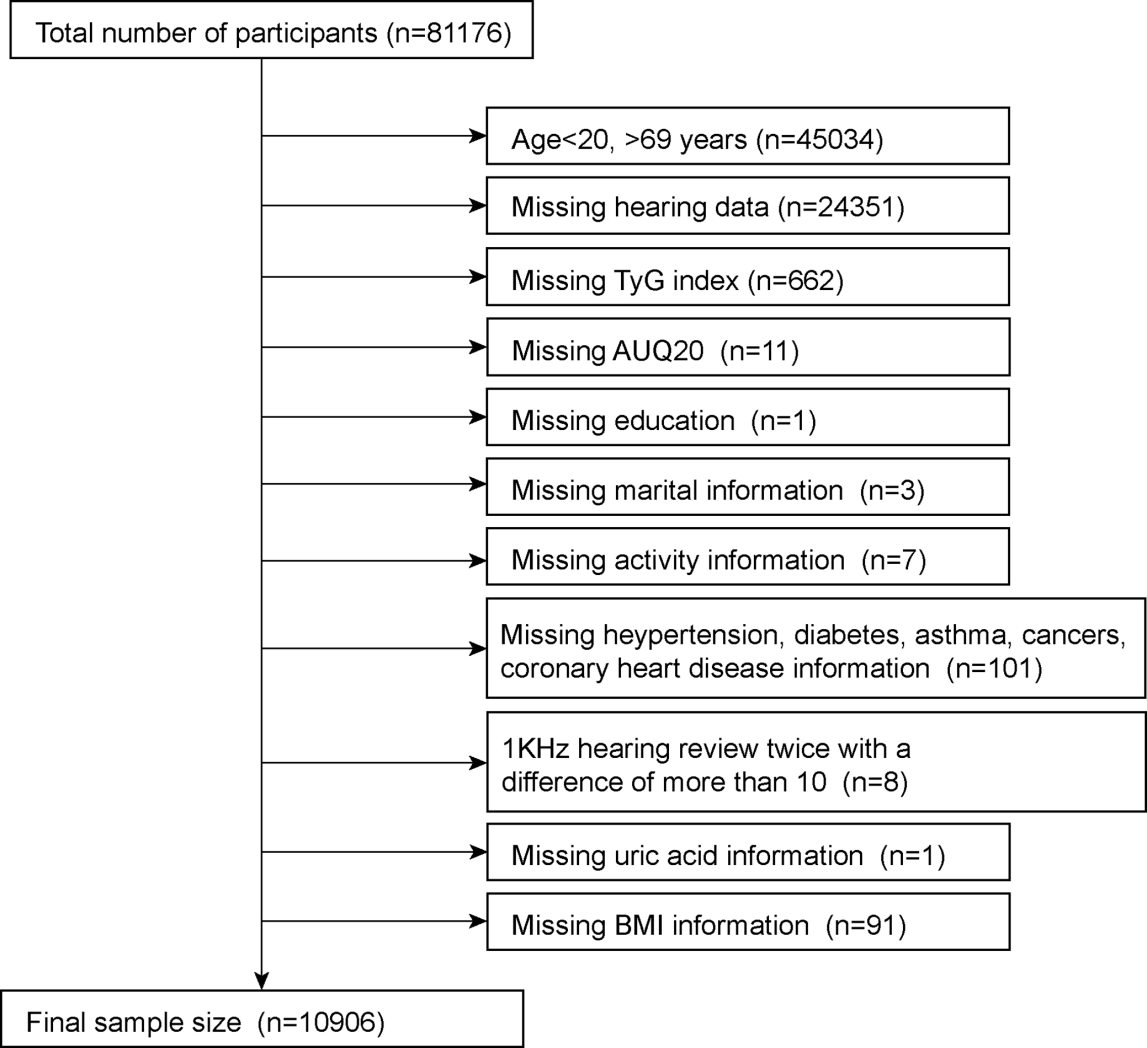
Sample selection process flow chart.

This study analyzed eight NHANES survey cycles (2001–2012, 2015–2018) to determine the number of hearing tests performed. This survey was filled out by 81,176 respondents. Since hearing was only tested in people aged 20–69 years, we first excluded those outside of this age group (n = 45,034). Subsequently, we removed participants who were missing information on their hearing data (n=24351) and TyG index (n=662). Meanwhile, we removed participants whose 1kHZ hearing test results differed by more than 10dB between the two tests (n=8). In the self-report questionnaire, we removed participants with missing information on various diseases, including hypertension, diabetes, coronary heart disease, asthma and cancer (n=101). Finally, participants with a small amount of missing information were also removed; these included AUQ20 (Participants were asked if they had a cold, sinus infection, or earache in the past 24 hours) (n=11), education (n=1), marital status (n=3), activity information (n=7), blood uric acid (n=1) and BMI (n=91). After excluding missing data, a total of 10,906 were included in the final study, with the specific exclusion criteria shown in **Figure 1**.

### 2.2 Data collection and definition

A triglyceride-glucose index (TyG) was designed as an exposure variable. TyG = ln [fasting triglycerides (mg/dl) *fasting glucose (mg/dl)/2]. An automated biochemistry analyzer was used to determine triglyceride levels and fasting glucose levels enzymatically. Using the Roche Cobas 6000 chemistry analyzer and the Roche Modular P, serum triglyceride concentrations were measured. Using a standardized protocol supplied by the NCHS, audiometry was performed on all eight cycles by trained examiners in a soundproof room at the mobile examination center. Both the beginning and the end of the field tests used the same audiometer calibrated to the same specification. Each ear’s G-conductivity threshold was measured over a range of 10 kHz to 120 kHz at 0.5, 1, 2, 3, 4, 6, and 8 kHz. A 1 kHz frequency was tested twice in each ear, and if the results differed by more than 10 dB, the participant was excluded (20), and the first response of the participant was analyzed (20, 21). LPTA (low frequency threshold) and HPTA (high frequency threshold) are the two categories of hearing, according to previous research. In LPTA, frequencies of 0.5, 1, and 2 kHz were averaged, while in HPTA, frequencies of 3, 4, 6, and 8 kHz were averaged. A PTA of greater than 15 dB in either ear was considered an outcome variable in this study. Low-frequency and high-frequency hearing loss were separated into these categories (21). A more stringent hearing impairment is considered to exist when there is simultaneous hearing impairment in both ears, with a PTA of >15 dB at low and high frequencies in both ears. Responses to the AUQ 20 prompted participants to state whether they had a cold, sinusitis, or earache within the past 24 hours.

Based on previous research (22–24), a multivariate-adjusted model was developed to summarise the potential confounding factors. We used sex, age, race, education level, poverty income ratio, marital status, alcohol consumption, physical activity, cholesterol, uric acid, smoking status, AUQ 20, hypertension, diabetes, coronary heart disease, cancer, energy intake, fat intake, sugar intake, and water intake as covariates for our study. Unless 2001–2002, all participants were asked to take two 24-hour dietary recalls; we will use the mean consumption of those two recalls for our analysis. We refer to previous studies (25–27) for specific information about the methods used to obtain these variables. At www.cdc.gov/nchs/nhanes/, you can find detailed measurements of all study variables.

### 2.3 Statistical methods

It is recommended that all statistical analyses be conducted using the NHANES sampling weights, stratifications, and clusterings provided in the study. To determine the weight associated with the variable to be studied in the maximum population, it is first necessary to specify the variable to be studied in the population. According to the weit selection guidelines, in this study, fasting triglyceride data were used, and the sub-weights corresponding to fasting triglycerides (WTSAF2YR) were divided by 8 for the final weight (26). By using the R language, we carried out a weighted analysis using the survey design R package. Weighted survey means and 95% CI are expressed for continuous variables, and weighted survey means and 95% CI are expressed for categorical variables. To assess differences between two groups, survey-weighted linear regression and survey-weighted chi-squared tests were used for continuous variables and survey-weighted chi-squared tests for categorical variables. Using multiple logistic regression models, three different types of models were examined to examine the prevalence of the TyG index and HI. A covariate adjustment was not made in Model 1. Several factors were taken into account in Model 2, such as ethnicity, marital status, and level of education. A complete model 3 was constructed by adjusting for all variables. In order to further assess the relationship between TyG index and HI, smooth curve fitting (the penalized spline method) and a generalized additive model (GAM) regression method were employed. When non-linear relationships were identified, the natural ratio test was used to determine the inflection point value. We then performed multiple regression analysis, taking into account sex, age, race, hypertension, and diabetes. A p value of 0.05 was considered statistically significant. In all analyses, Empower Stats was used (www.empowerstats.com; X&Y Solutions, Inc., Boston, MA, USA) as well as R 4.0.2 (http://www.R-project.org, The R Foundation). Those numerical variables that had more missing values were converted to categorical variables, with the lowest quantile as the reference group, and evaluated from the median to the interquartile range.

## 3 Results

### 3.1 Baseline characteristics

The baseline demographic characteristics of the included participants were shown in **Table 1**. A total of 10906 participants were included in this study, 5313 of whom were men (48.72%). Weighted characteristics were subdivided according to TyG tertiles (Q1: 5.75-8.30, Q2: 8.30-8.92,Q3:8.92-13.25). In addition to PIR, alcohol consumption, and asthma, there were significant differences in baseline characteristics between different TyG tertiles. Individuals in the highest TyG tertiles tended to have an older age, higher cholesterol, higher uric acid, a higher BMI, diabetes, hypertension, coronary heart disease, high-frequency hearing impairment, severe high-frequency hearing impairment, and the highest number of smokers.

**Table 1:** Baselines characteristics of participants, weighted.

TyG Index	Q1 (5.75-8.30)	Q2 (8.30-8.92)	Q3 (8.92-13.25)	P-value
Age (years)	39.46 (38.62,40.30)	43.75 (43.02,44.49)	46.93 (46.25,47.61)	<0.0001
Serum Cholesterol (mg/dl)	180.99 (179.47,182.51)	198.10 (196.44,199.76)	213.86 (211.05,216.67)	<0.0001
BMI (kg/m2)	26.37 (26.04,26.70)	29.25 (28.90,29.60)	31.24 (30.90,31.57)	<0.0001
Serum Uric Acid (mg/dl)	4.90 (4.84,4.96)	5.39 (5.33,5.44)	5.81 (5.73,5.89)	<0.0001
Gender (%)				<0.0001
Male	41.08 (39.22,42.96)	48.58 (46.43,50.74)	59.31 (56.75,61.82)	
Female	58.92 (57.04,60.78)	51.42 (49.26,53.57)	40.69 (38.18,43.25)	
Race (%)				<0.0001
Mexican American	10.86 (9.00,13.06)	14.76 (12.00,18.03)	17.57 (13.93,21.91)	
White	66.12 (62.11,69.91)	69.13 (64.87,73.09)	66.71 (61.63,71.43)	
Black	15.59 (12.82,18.82)	9.77 (7.82,12.16)	6.96 (5.51,8.77)	
Other Race	7.43 (6.28,8.77)	6.33 (5.25,7.62)	8.76 (7.15,10.69)	
Education Level (%)				<0.0001
Less than high school	10.97 (9.36,12.83)	14.80 (12.60,17.31)	16.53 (14.35,18.96)	
High school	19.40 (17.52,21.43)	22.42 (20.57,24.38)	22.33 (20.12,24.71)	
More than high school	69.63 (66.57,72.52)	62.78 (59.52,65.93)	61.14 (57.78,64.40)	
Marital Status (%)				<0.0001
Cohabitation	61.73 (58.49,64.86)	66.28 (63.85,68.63)	69.58 (67.21,71.86)	
Solitude	38.27 (35.14,41.51)	33.72 (31.37,36.15)	30.42 (28.14,32.79)	
Alcohol(%)				0.3471
Yes	73.94 (71.18,76.52)	74.44 (72.22,76.54)	74.92 (72.80,76.93)	
No	20.41 (18.35,22.63)	20.29 (18.40,22.32)	20.79 (19.00,22.70)	
Unclear	5.65 (4.37,7.28)	5.27 (4.25,6.51)	4.29 (3.40,5.39)	
High Blood Pressure (%)				<0.0001
Yes	16.85 (15.29,18.55)	27.22 (25.37,29.14)	36.09 (33.59,38.68)	
No	83.15 (81.45,84.71)	72.78 (70.86,74.63)	63.91 (61.32,66.41)	
Diabetes (%)				<0.0001
Yes	2.46 (1.87,3.24)	4.94 (3.98,6.12)	16.24 (14.49,18.16)	
No	97.54 (96.76,98.13)	95.06 (93.88,96.02)	83.76 (81.84,85.51)	
Smoked (%)				<0.0001
Yes	38.68 (36.35,41.07)	47.71 (45.17,50.26)	50.76 (48.83,52.68)	
No	61.32 (58.93,63.65)	52.29 (49.74,54.83)	49.24 (47.32,51.17)	
Physical Activity (%)				<0.0001
Never	21.21 (19.45,23.08)	25.98 (23.76,28.33)	30.83 (28.24,33.55)	
Moderate	27.38 (25.40,29.45)	31.71 (29.61,33.88)	32.18 (29.91,34.53)	
Vigorous	51.41 (48.94,53.87)	42.31 (39.95,44.71)	36.99 (34.28,39.79)	
Asthma (%)				0.4374
Yes	15.13 (13.75,16.61)	15.45 (13.60,17.49)	13.91 (12.01,16.05)	
No	84.87 (83.39,86.25)	84.55 (82.51,86.40)	86.09 (83.95,87.99)	
Coronary Artery Disease (%)				<0.0001
Yes	1.27 (0.86,1.87)	1.71 (1.19,2.45)	3.44 (2.71,4.36)	
No	98.73 (98.13,99.14)	98.29 (97.55,98.81)	96.56 (95.64,97.29)	
Cancers (%)				0.0002
Yes	5.17 (4.22,6.31)	6.98 (5.89,8.27)	9.07 (7.67,10.71)	
No	94.83 (93.69,95.78)	93.02 (91.73,94.11)	90.93 (89.29,92.33)	
AUQ020 (%)				0.3894
Yes	11.69 (10.20,13.38)	13.00 (11.70,14.42)	12.76 (11.26,14.43)	
No	88.31 (86.62,89.80)	87.00 (85.58,88.30)	87.24 (85.57,88.74)	
Low-frequency HI (%)				<0.0001
No	80.62 (78.38,82.68)	71.28 (69.06,73.40)	65.79 (63.45,68.06)	
Yes	19.38 (17.32,21.62)	28.72 (26.60,30.94)	34.21 (31.94,36.55)	
High-frequency HI (%)				<0.0001
No	52.25 (49.06,55.42)	38.13 (35.38,40.97)	27.05 (24.80,29.42)	
Yes	47.75 (44.58,50.94)	61.87 (59.03,64.62)	72.95 (70.58,75.20)	
Serious low-frequency HI (%)				<0.0001
No	90.97 (89.41,92.32)	84.12 (81.90,86.11)	79.68 (77.78,81.46)	
Yes	9.03 (7.68,10.59)	15.88 (13.89,18.10)	20.32 (18.54,22.22)	
Serious high-frequency HI (%)				<0.0001
No	68.32 (65.57,70.95)	55.33 (52.37,58.27)	43.03 (39.91,46.20)	
Yes	31.68 (29.05,34.43)	44.67 (41.73,47.63)	56.97 (53.80,60.09)	
PIR (%)				0.2638
<1.3	19.34 (16.84,22.11)	19.19 (17.23,21.32)	20.75 (18.23,23.51)	
≥1.3<3.5	31.61 (29.10,34.24)	32.80 (30.24,35.46)	33.12 (30.52,35.82)	
≥3.5	43.75 (40.30,47.26)	42.02 (38.59,45.53)	39.75 (35.80,43.85)	
Unclear	5.30 (4.35,6.43)	5.99 (4.76,7.51)	6.38 (5.22,7.77)	
Total Kcal (%)				0.0093
Lower	43.21 (41.02,45.43)	42.82 (40.19,45.50)	37.82 (34.71,41.02)	
Higher	44.88 (42.84,46.94)	45.49 (42.92,48.08)	50.26 (47.22,53.31)	
Unclear	11.91 (10.48,13.51)	11.69 (10.40,13.13)	11.92 (10.46,13.55)	
Total Sugar (%)				0.1087
Lower	41.02 (39.14,42.93)	38.23 (36.17,40.32)	38.36 (36.38,40.38)	
Higher	38.17 (36.44,39.93)	39.40 (37.34,41.50)	41.01 (39.10,42.95)	
Unclear	20.81 (19.00,22.74)	22.37 (20.55,24.30)	20.63 (19.09,22.26)	
Total Water (%)				0.0981
Lower	42.68 (40.54,44.85)	44.06 (41.07,47.09)	39.62 (36.91,42.40)	
Higher	45.41 (43.32,47.51)	44.25 (41.29,47.25)	48.46 (45.71,51.22)	
Unclear	11.91 (10.48,13.51)	11.69 (10.40,13.13)	11.92 (10.46,13.55)	
Total Fat (%)				0.004
Lower	41.88 (39.78,44.01)	43.07 (40.59,45.59)	37.45 (34.88,40.10)	
Higher	46.21 (44.14,48.29)	45.23 (42.81,47.68)	50.63 (48.03,53.22)	
Unclear	11.91 (10.48,13.51)	11.69 (10.40,13.13)	11.92 (10.46,13.55)	

For categorical variables: survey-weighted percentage (95% CI), P-value was by survey-weighted Chi-square test (svytable).

AUQ020:Participants were asked if they had a cold, sinus infection, or earache in the past 24 hours.

### 3.2 A higher TyG index was associated with a higher prevalence of high-frequency HI, but not with low-frequency HI

It was observed that the TyG index was positively associated with the increased prevalence of high-frequency HI. Based upon the fully adjusted model (model 3) of the study, this strong association with high-frequency HI remained stable (OR = 1.12, 95% CI: 1.03, 1.22), showing that an increase in TyG led to an increase in the risk of high-frequency HI by 12%. To analyze sensitivity, we converted the TyG index from a continuous variable to a categorical variable (tertiles). A 25% increase in tertile 3 was observed when compared with the lowest TyG index tertile (tertile 1) (OR = 1.25, 95% CI: 1.09, 1.44), as shown in **Table 2**. In **Supplementary Table 1**, the TyG index correlated positively with low-frequency HI prevalence, but this relationship was not statistically significant.

**Table 2:** Logistic regression analysis between TyG index with high-frequency HI prevalence.

Characteristic	Model 1 OR (95%CI)	Model 2 OR (95%CI)	Model 3 OR (95%CI)
High-frequency HI			
TyG Index	1.95 (1.84, 2.06)	1.19 (1.11, 1.27)	1.12 (1.03, 1.22)
Categories			
Tertile 1 (Lower)	1	1	1
Tertile 2 (Middle)	1.78 (1.62, 1.96)	1.16 (1.03, 1.31)	1.11 (0.98, 1.25)
Tertile 3 (Higher)	3.00 (2.72, 3.30)	1.38 (1.22, 1.57)	1.25 (1.09, 1.44)
P for trend	< 0.001	< 0.05	< 0.05
Serious high-frequency HI			
TyG Index	1.91 (1.81, 2.02)	1.22 (1.14, 1.30)	1.14 (1.05, 1.24)
Categories			
Tertile 1 (Lower)	1	1	1
Tertile 2 (Middle)	1.82 (1.66, 2.01)	1.19 (1.05, 1.35)	1.13 (0.99, 1.28)
Tertile 3 (Higher)	3.00 (2.73, 3.31)	1.43 (1.26, 1.62)	1.27 (1.10, 1.46)
P for trend	< 0.001	< 0.05	< 0.05

Model 1, was adjusted for no covariates;

Model 2, was adjusted for age,gender, race,marital status and education;

Mode 3, adjusted for all covariates except effect modifier.

### 3.3 Dose response and threshold effect analysis of TyG index on high-frequency HI prevalence

**Figure 2:**
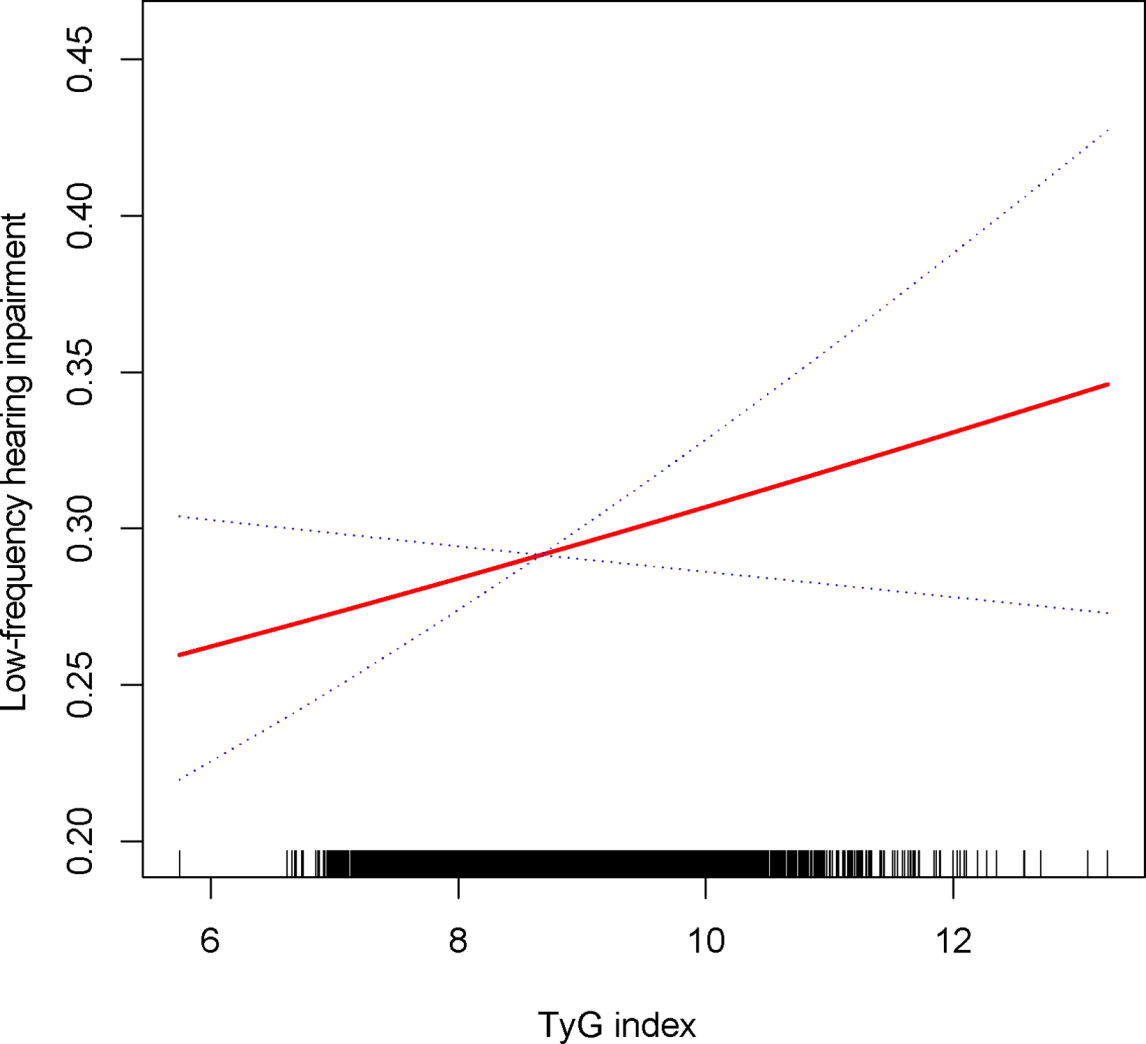
Density dose-response relationship between TyG with low-frequency HI. The area between the upper and lower dashed lines is represented as 95% CI. Each point shows the magnitude of the TyG and is connected to form a continuous line. Adjusted for all covariates except effect modifier.

**Figure 3:**
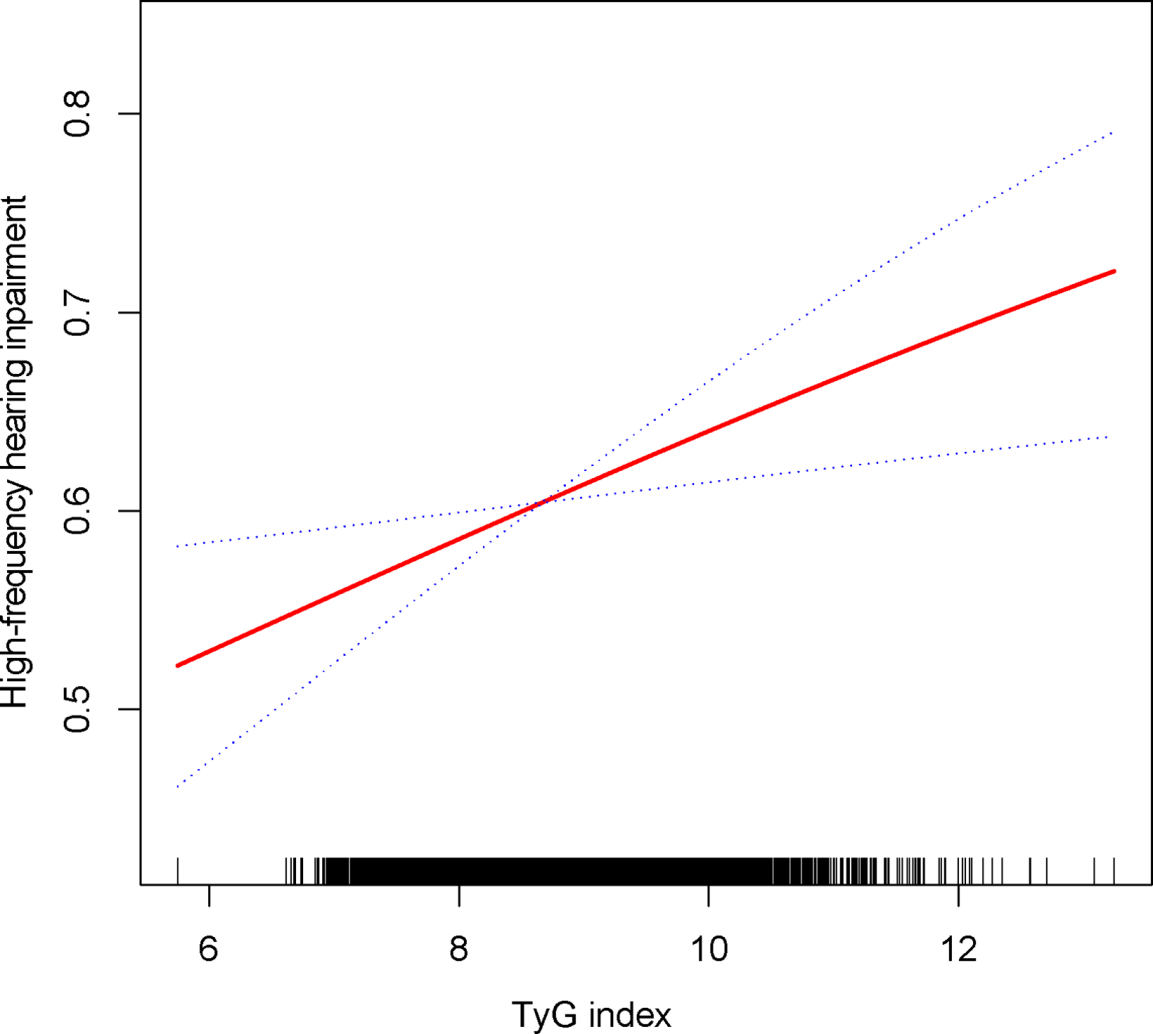
Density dose-response relationship between TyG with high-frequency HI.The area between the upper and lower dashed lines is represented as 95% CI. Each point shows the magnitude of the TyG and is connected to form a continuous line. Adjusted for all covariates except effect modifier.

The relationship between the TyG index and the prevalence of HI was further explored using a generalized additive model and smooth curve fitting. Our results indicate a linear positive correlation between the TyG index and the prevalence of HI, with both low-frequency and high-frequency HI (**Figures 2**, **3**).

### 3.4 Subgroup analysis

Subgroup analyses were conducted to examine the robustness of the association between the TyG index and high-frequency HI prevalence. Results: females (OR = 1.14,95%CI: 1.02,1.29), age <40 years (OR = 1.18,95%CI: 1.05,1.32), age 40-69 years (OR = 1.14,95%CI: 1.02,1.27), non-hypertension (OR = 1.12,95%CI: 1.01,1.23), non-diabetics (OR = 1.13,95%CI: 1.04,1.24) (**Table 3**).

**Table 3:** Subgroup analysis between TyG index with high-frequency HI prevalence.

Characteristic	Model 1 OR (95%CI)	Model 2 OR (95%CI)	Model 3 OR (95%CI)
High-frequency HI			
Stratified by gender			
Male	1.70 (1.57, 1.85)	1.16 (1.05, 1.28)	1.11 (0.99, 1.26)
Female	2.05 (1.89, 2.22)	1.22 (1.11, 1.35)	1.14 (1.02, 1.29)
Stratified by race			
Mexican American	2.04 (1.82, 2.28)	1.13 (0.99, 1.30)	1.05 (0.89, 1.23)
White	2.05 (1.85, 2.26)	1.26 (1.12, 1.42)	1.09 (0.94, 1.26)
Black	1.79 (1.58, 2.02)	1.12 (0.97, 1.29)	1.15 (0.97, 1.36)
Other Race	1.94 (1.67, 2.25)	1.21 (1.01, 1.46)	1.19 (0.96, 1.49)
Stratified by age(years)			
20-39	1.55 (1.41, 1.70)	1.34 (1.22, 1.48)	1.18 (1.05, 1.32)
40-69	1.55 (1.42, 1.70)	1.36 (1.24, 1.50)	1.14 (1.02, 1.27)
Stratified by hypertension			
Yes	1.51 (1.34, 1.69)	1.11 (0.97, 1.27)	1.09 (0.93, 1.28)
No	1.89 (1.77, 2.02)	1.20 (1.11, 1.30)	1.12 (1.01, 1.23)
Stratified by diabetes			
Yes	1.14 (0.94, 1.38)	0.98 (0.79, 1.22)	1.11 (0.86, 1.44)
No	1.85 (1.74, 1.97)	1.17 (1.09, 1.27)	1.13 (1.04, 1.24)
Serious high-frequency HI			
Stratified by gender			
Male	1.66 (1.54, 1.79)	1.21 (1.10, 1.33)	1.17 (1.04, 1.31)
Female	2.06 (1.90, 2.24)	1.25 (1.13, 1.38)	1.14 (1.01, 1.28)
Stratified by race			
Mexican American	1.89 (1.70, 2.10)	1.12 (0.99, 1.28)	1.07 (0.91, 1.24)
White	2.04 (1.85, 2.24)	1.29 (1.15, 1.46)	1.16 (1.01, 1.34)
Black	1.79 (1.59, 2.02)	1.16 (1.00, 1.34)	1.12 (0.95, 1.33)
Other Race	1.98 (1.70, 2.30)	1.30 (1.08, 1.57)	1.25 (0.99, 1.56)
Stratified by age(years)			
20-39	1.63 (1.45, 1.82)	1.38 (1.22, 1.55)	1.13 (0.97, 1.31)
40-69	1.51 (1.41, 1.63)	1.37 (1.27, 1.48)	1.17 (1.07, 1.28)
Stratified by hypertension			
Yes	1.53 (1.38, 1.69)	1.26 (1.12, 1.43)	1.19 (1.03, 1.37)
No	1.87 (1.75, 2.00)	1.18 (1.08, 1.29)	1.12 (1.01, 1.24)
Stratified by diabetes			
Yes	1.17 (1.01, 1.36)	1.12 (0.94, 1.35)	1.15 (0.93, 1.42)
No	1.82 (1.71, 1.93)	1.18 (1.09, 1.28)	1.14 (1.05, 1.25)

Model 1, no covariates were adjusted.

Model 2, Model 1+age,gender, race,marital status and education;

Mode 3, adjusted for all covariates except effect modifier.

### 3.5 Sensitivity analysis

**Figure 4:**
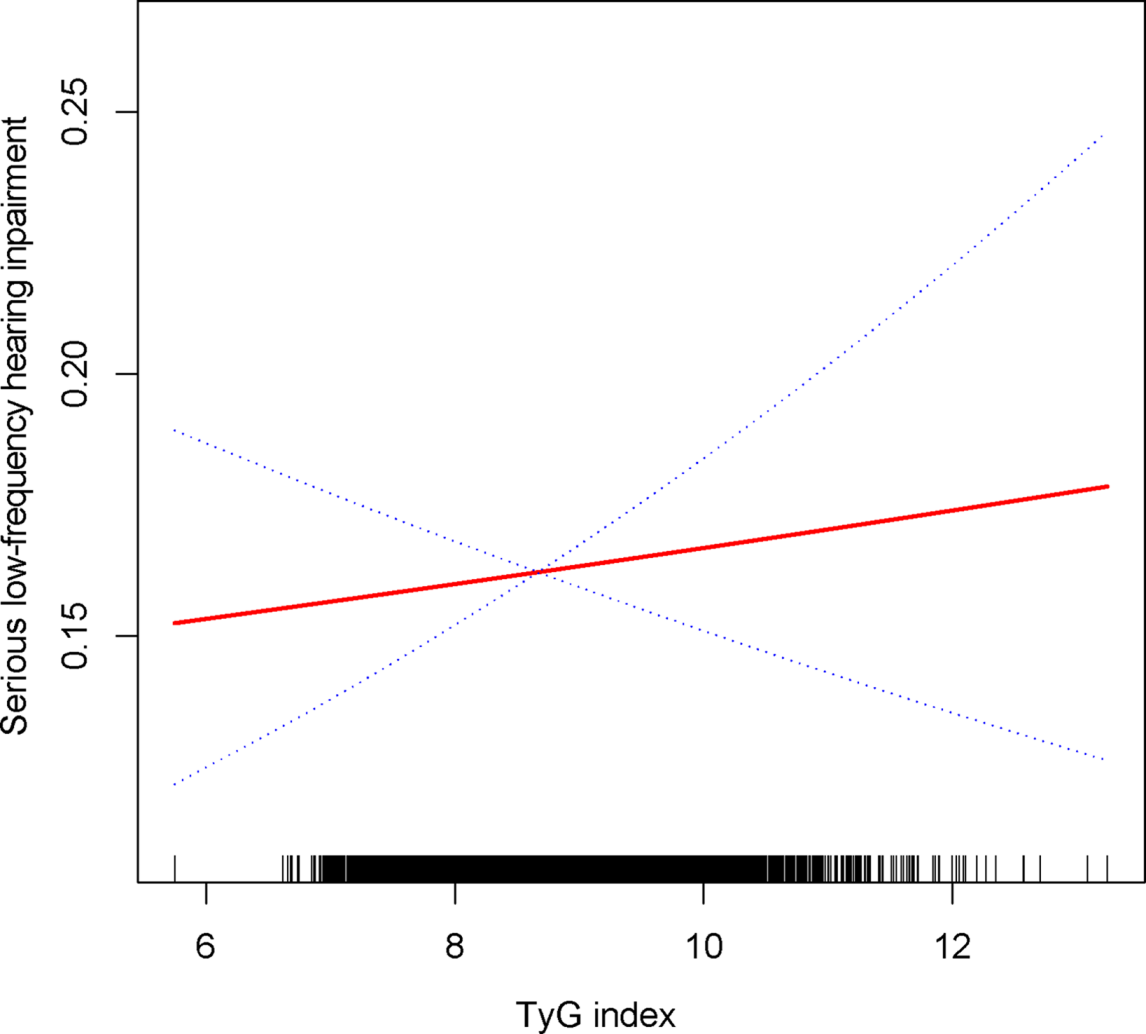
Density dose-response relationship between TyG with serious low-frequency HI. The area between the upper and lower dashed lines is represented as 95% CI. Each point shows the magnitude of the TyG and is connected to form a continuous line. Adjusted for all covariates except effect modifier.

**Figure 5:**
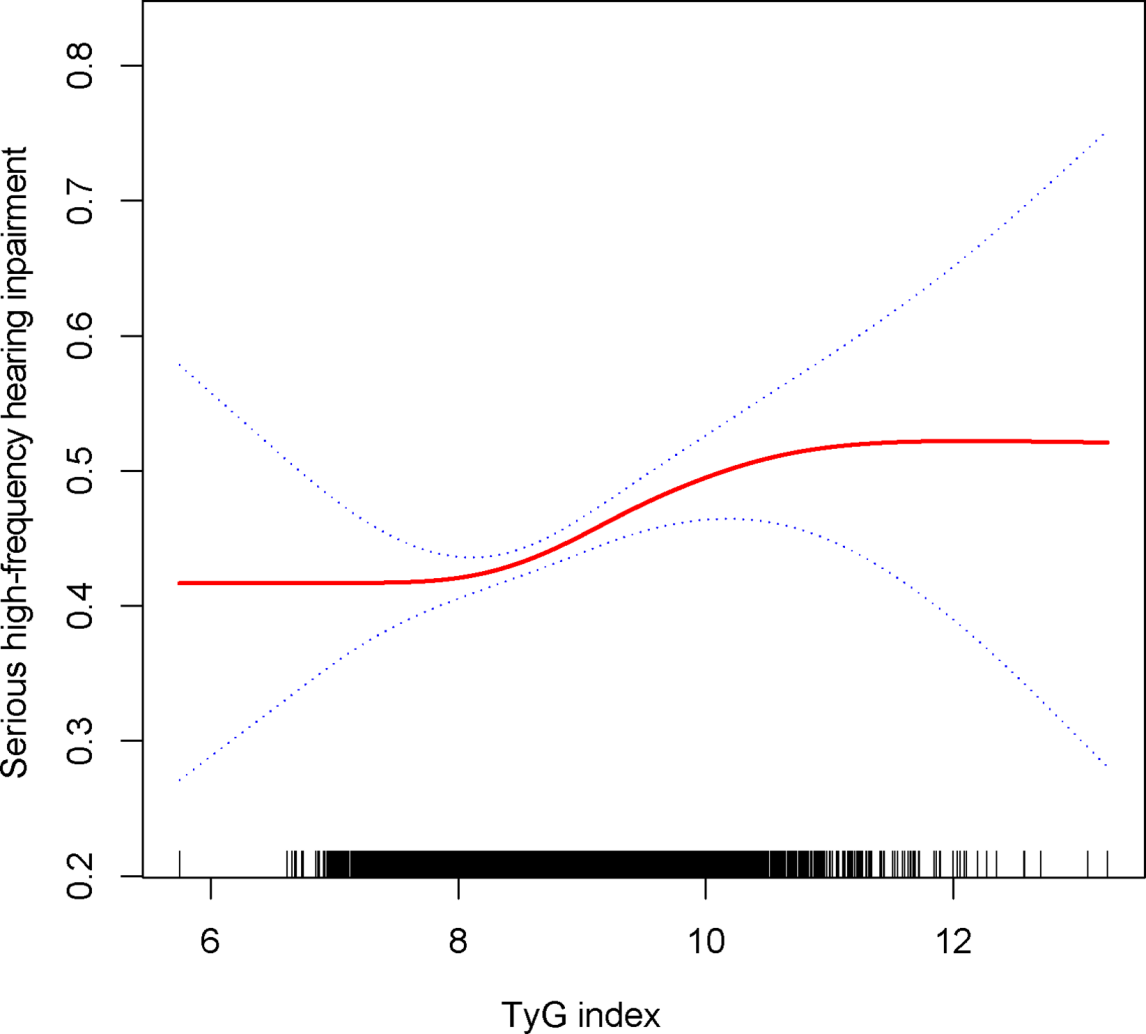
Density dose-response relationship between TyG with serious high-frequency HI. The area between the upper and lower dashed lines is represented as 95% CI. Each point shows the magnitude of the TyG and is connected to form a continuous line. Adjusted for all covariates except effect modifier.

TyG index was still associated with a higher prevalence of severe high frequency HI among those with PTAs greater than 15 dB in both ears (OR = 1.14, 95% CI: 1.05, 1.24) when using a stricter definition of HI (**Table 2**). In tertile 3, the odds of experiencing severe hearing loss increased significantly by 27% when the TyG index was divided into three scores (OR = 1.27, 95% CI: 1.10, 1.46) (**Table 2**). In **Supplementary Table 1**, we found that although the TyG index was positively correlated with serious low-frequency HI, it was still not statistically different, and the smooth curve fit suggested a linear effect between the independent variable and the response variable (**Figure 4**). TyG index was found to have a non-linear positive association with severe high-frequency HI with a generalised additive model and smooth curve fitting (**Figure 5**), but saturation threshold effect analysis failed to find an appropriate inflection point value (**Supplementary Table 2**). The following stratified analysis showed that male group (OR = 1.17, 95% CI: 1.04, 1.31), female group (OR = 1.14, 95% CI: 1.01, 1.28), white group (OR = 1.16, 95% CI: 1.01, 1.34), age group 40-69 years (OR = 1.17, 95% CI: 1.07, 1.28), hypertensive group (OR = 1.19, 95%CI: 1.03, 1.37), non-hypertensive group (OR = 1.12, 95%CI: 1.01, 1.24), non-diabetic group (OR = 1.14, 95%CI: 1.05, 1.24) (**Table 3**).

## 4 Discussion

According to Seo M., mild high-frequency hearing impairment may be associated with HOMA-IR in the Korean population (11), and there are no other reports that link the IR correlation index to such hearing impairment. It is the first cross-sectional study to show a correlation between the TyG index and the health index. The HI and TyG indexes were initially defined as having a positive correlation in this study. The correlation between low-frequency HI and high-frequency HI was also positive, but the statistical difference was not readily apparent. Similar to previous studies (11), we found a higher TyG index was associated with high-frequency HI.

Diabetes mellitus is a well established risk factor for HI, but the exact mechanism by which diabetes mellitus causes the development of HI remains unclear (28). Researchers have suggested that diabetes-induced microangiopathy and peripheral neuropathy might contribute to HI (29, 30). However, IR, a high risk factor for diabetes, has been shown in many studies to be at risk for autonomic neuropathy in patients with pre-defined diabetes, such as cardiac vagal dysfunction (31), respiratory dysfunction (32) and gastrointestinal motility disorders (33). One study showed that cochlear dysfunction due to this peripheral neuropathy was mainly associated with chronic hyperglycaemia and not with acute hyperglycaemia (34). This might therefore underline the possible role of TyG index in diabetic patients.

Diabetes mellitus is a well established risk factor for HI, but the exact mechanism by which diabetes mellitus causes the development of HI remains unclear (28). Researchers have suggested that diabetes-induced microangiopathy and peripheral neuropathy might contribute to HI (29) (30). However, IR, a high risk factor for diabetes, has been shown in many studies to be at risk for autonomic neuropathy in patients with pre-defined diabetes, such as cardiac vagal dysfunction (31), respiratory dysfunction (32), and gastrointestinal peristalsis (33). One study showed that cochlear dysfunction due to this peripheral neuropathy was mainly associated with chronic hyperglycaemia and not with acute hyperglycaemia (34). This might therefore underline the possible role of TyG index in diabetic patients. Additionally, although there are few mechanistic studies of how IR causes HI, IR has been shown to be closely linked to obesity (35), and obesity has been linked to HI. Capillary wall tension caused by excess adipose tissue may be responsible for damaging the fragile inner ear system (36). In studies that tested IR using the TyG index, which is a composite of fasting triglyceride (TG) and fasting glucose (FG) levels, it was shown to be better than the HOMA-IR index because it is insulin-independent (14, 16, 36). A high blood glucose level can lead to a number of adverse changes in the body (37), including mitochondrial destruction associated with abnormal oxidative phosphorylation and an inhibited production of ATP. A hyperglycemic patient is more likely to be diagnosed with kidney or inner ear dysfunction as a result of the above pathophysiological changes caused by hyperglycemia (38, 39). When mitochondria fail, the vasculature of the inner ear is one of the primary sites of injury, and elevated blood glucose will ultimately cause marginal cell damage and necrosis (40). As previously reported (41, 42), HI is associated with increased triglycerides and decreased High-density lipoprotein (HDL). In spite of the fact that the underlying mechanisms of triglyceride-induced HI and HI pathogenesis are still unclear, altered microcirculation in the inner ear may have a profound effect on these conditions. As well as structural changes to blood vessels, physicochemical changes of blood and hemodynamic changes of blood also affect blood supply (43). It has been discovered that abnormal triglyceride levels can lead to alterations in the viscosity of whole blood, which may be responsible for hemodynamic changes in the inner ear, which may explain the absence of sufficient blood supply to target organs (44).

We found a positive correlation between IR and high-frequency HI (including strict HI) in this study, as well as sensitive populations that require further attention. In the HPTA test, the most sensitive population for HI in both ears was female participants between 20 and 39 years of age, while the most sensitive population for HI in both ears in the HPTA was males between 40 and 69 years of age. In previous studies, researchers found that there was a difference in the probability of HI occurring in each ear before and after a certain age group between males and females (45). For people aged 40–50, the probability of hearing impairment in both ears is similar, and the sex with the highest chance of hearing impairment at this age is female or male (45). It is not known whether HI is more likely in males or females before the age of 50, but it is more likely after the age of 50 (45). In previous studies, oestradiol has been found to protect hearing (46, 47), but some studies (48) have indicated that oral oestradiol increases the risk of hearing loss in menopausal women and that the duration of oral oestradiol is clearly correlated with HI risk. Meanwhile, almost all researchers agree that progesterone can cause hearing loss (48, 49), which is attributed to an increase in blood vessel inflammation in the inner ear (50). By contrast, androgens present in young men are beneficial to hearing (51, 52). With increased age, women, however, tend to have better hearing than men. Several studies suggest that noise exposure and other ototoxic injuries are more prevalent in men as they age (51). Additionally, estrogen is a growth factor hormone that contributes to cell proliferation in various body systems (including the inner ear), in addition to being an important part of the ovulation cycle and pregnancy. It is therefore not surprising that female organisms (mammals) have better hearing with age than male organisms (53). Our findings suggest that the effects of age and gender on monoaural and binaural HI may be due to hormonal changes, but the exact mechanism is still unknown. Among participants with diabetes and hypertension, we were surprised to find that binaural HI was more likely to occur in participants with diabetes and hypertension. However, the positive association between the TyG index and monaural HI was stronger in participants without diabetes or hypertension. In addition to diabetes mellitus, high blood pressure is a relatively well-documented risk factor for having HI as well. However, the relationship between hearing loss and diabetes remains controversial. According to several studies, diabetes does not cause hearing loss (54–56), and a longitudinal study found that diabetes causes generalized hearing loss but not incidental hearing loss (57). In a cohort study, Kim MB found a positive association between diabetes and bilateral ear HI. We did not deny that diabetes is an important factor affecting HI, but our results remind those without diabetes to take precautions. Participants with diabetes should pay greater attention to the possibility that a higher TyG index may lead to unilateral HI, and a higher TyG index may lead to bilateral HI as well. Although hypertension is a typical vascular disease that theoretically increases the risk of HI, the evidence does not support this claim. According to Padilha FYOMM and Samelli AG, hypertensive people were at an increased risk of HI, but after adjusting for other factors, hypertension did not significantly increase HI risk (58, 59). According to Lin BM, hypertension increases the risk of HI by only a small amount (60). It is therefore necessary to investigate the characteristics of the positive correlation between the TyG index and HI in the hypertensive population. The present results suggest that HI with a higher TyG index may be more prevalent in non-hypertensive populations, while the positive correlation between TyG index and HI with bilateral ears may be more common in hypertensive populations. Dąbrowski M demonstrated that a higher degree of urinary albumin excretion was associated with HI (61). Hypertension is a common comorbidity, particularly in patients with metabolic dysfunction, such as type 2 diabetes mellitus. We found a higher risk of binaural HI in participants with hypertension and diabetes, which was similar to the results of Dąbrowski M. Several classes of drugs have been shown to improve urinary albumin excretion and improve clinical outcomes in patients with type 2 diabetes, whether or not these patients have hypertension (62). This could potentially have an impact on sensitive populations who had a higher risk of developing monaural HI in our study.

Considering the sample size, this is the first cross-sectional study to demonstrate a positive relationship between the TyG index and HI. However, our study has several limitations. In the first place, cross-sectional studies cannot be used to prove causality, and prospective studies are required to further confirm the findings. Second, as a cross-sectional study, we included as many confounding factors as possible and adjusted them in the model to better prove the independent effect between independent and variable variables, but there are still other potential confounding factors. Moreover, although NHANES participants are representative, the proportion of HI in both ears is still low, and fewer individuals have severe HI, so the current conclusions may not be appropriate for this group. Finally, due to the lack of validation of gold standard hyperinsulinemic-hyperglycaemic clamp test data, our current results would still need to be validated by subsequent studies.

## 5 Summary

TyG index correlates linearly with HI, particularly among high-frequency HI participants, which applies to unilateral and bilateral HI. Women aged 20-39, without diabetes or hypertension, were more likely to be associated with unilateral HI based on TyG, whereas men aged 40-69 years, with diabetes and hypertension, may be more likely to develop bilateral HI if their TyG index is higher.

## Data availability statement

The raw data supporting the conclusions of this article will be made available by the authors, without undue reservation.

## Ethics statement

The NCHS Research Ethics Review Committee approved the NHANES survey protocol (https://www.cdc.gov/nchs/nhanes/irba98.htm), and all participants of the study provided informed written consent. The NHANES database is open to the public and therefore the ethical review of this study was exempt.

## Author contributions

Data analysis and manuscript writing: LL. Study design and statistical advice: LL, MQ and JJ. Manuscript editing: MQ, JJ and WW. Validation and review: WW, LL, MQ and JJ. Quality control: WW. All authors contributed to the article and approved the submitted version.

## Supplementary material

Supplementary Table 1 Logistic regression analysis between TyG index with low-frequency HI prevalence. Model 1=no covariates were adjusted. Model 2=Model 1+age, gender, race, marital status and education; Mode3=adjusted for all covariates.

Supplementary Table 2 Two Piece-Wise Linear Regression and Logarithmic Likelihood Ratio Test Explain Threshold Effect Analysis of TyG Index with serious high-frequency HI Prevalence. ULR,univariate linear regression;PLR,piecewise linear regression;LRT,logarithmic likelihood ratio test,statistically significant:p<0.05.
