# Supplementary material for: Correlation between hearing impairment and the Triglyceride Glucose Index: based on a national cross-sectional study
Source: Front Endocrinol (Lausanne). 2023 Jun 22;14:1216718. doi: 10.3389/fendo.2023.1216718 (PMC10325635; doi:10.3389/fendo.2023.1216718)
Supplement: Supplementary Table 1 — Logistic regression analysis between TyG index with low-frequency HI prevalence. Model 1=no covariates were adjusted. Model 2=Model 1+age, gender, race, marital status and education; Mode3=adjusted for all covariates. [file Table_1.doc]

**Supplementary Table 1.**Logistic regression analysis between TyG index with low-frequency HI prevalence

| **Characteristic** | **Model 1 OR(95%CI)** | **Model 2 OR(95%CI)** | **Model 3 OR(95%CI)** |
| --- | --- | --- | --- |
| Low-frequency HI |  |  |  |
| TyG Index | 1.52 (1.44, 1.61) | 1.14 (1.07, 1.22) | 1.05 (0.98, 1.14) |
| Serious low-frequency HI |  |  |  |
| TyG Index | 1.54 (1.44, 1.64) | 1.14 (1.06, 1.23) | 1.03 (0.94, 1.12) |

Model 1=no covariates were adjusted.

Model 2=Model 1+age, gender, race, marital status and education;

Mode3=adjusted for all covariates.

**Supplementary Table 2.** Two Piece-Wise Linear Regression and Logarithmic Likelihood Ratio Test Explain Threshold Effect Analysis of TyG Index with serious high-frequency HI Prevalence

| **BRI Index** | **ULR Test** | **PLR Test** | **LRT test** |
| --- | --- | --- | --- |
|  | **β(95%CI)** | **β(95%CI)** | **P value** |
| ＜8.17 | 1.14 (1.05, 1.24) | 0.89 (0.67, 1.18) | 0.073 |
| ≥8.17 |  | 1.20 (1.09, 1.32) |  |

ULR,univariate linear regression;PLR,piecewise linear regression;LRT,logarithmic likelihood ratio test,statistically significant:p<0.05.
